# Dimerization Capacities of FGF2 Purified with or without Heparin-Affinity Chromatography

**DOI:** 10.1371/journal.pone.0110055

**Published:** 2014-10-09

**Authors:** Natalia Platonova, Géraldine Miquel, Liang-Yuan Chiu, Said Taouji, Elisabetta Moroni, Giorgio Colombo, Eric Chevet, Shih-Che Sue, Andreas Bikfalvi

**Affiliations:** 1 INSERM U1029, Allée Geoffroy St. Hilaire, Pessac, France; 2 Université Bordeaux I, Allée Geoffroy St. Hilaire, Pessac, France; 3 INSERM U1053, Team Avenir, Bordeaux, France; 4 Institute of Bioinformatics and Structure Biology, National Tsing Hua University, Hsinchu, Taiwan; 5 Istituto di Chimica del Riconoscimento Molecolare, CNR, Milano, Italy; IDI, Istituto Dermopatico dell'Immacolata, Italy

## Abstract

Fibroblast growth factor-2 (FGF2) is a pleiotropic growth factor exhibiting a variety of biological activities. In this article, we studied the capacity of FGF2 purified with or without heparin affinity chromatography to self-associate. Analyzing the NMR HSQC spectra for different FGF2 concentrations, heparin-affinity purified FGF2 showed perturbations that indicate dimerization and are a higher-order oligomerization state. HSQC perturbation observed with different FGF2 concentrations revealed a heparin-binding site and two dimer interfaces. Thus, with increasing protein concentrations, FGF2 monomers make contacts with each other and form dimers or higher order oligomers. On the contrary, FGF2 purified with ion-exchange chromatography did not show similar perturbation indicating that self-association of FGF2 is eliminated if purification is done without heparin-affinity chromatography. The HSQC spectra of heparin-affinity purified FGF2 can be reproduced to some extent by adding heparin tetra-saccharide to ion exchange chromatography purified FGF2. Heparin-affinity purified FGF2 bound to acceptor and donor beads in a tagged form using His-tagged or GST-tagged proteins, also dimerized in the AlphaScreen^™^ assay. This assay was further validated using different experimental conditions and competitors. The assay constitutes an interesting tool to study dimerization of other FGF forms as well.

## Introduction

Fibroblast growth factors (FGFs) are broad-range morphogens that have significant functional roles in early and late embryonic development. For example, genetic analyses in mice have demonstrated that FGFs play crucial roles in mesoderm induction and in lung and brain development [1], [2]. Furthermore, FGFs are thought to be implicated in renewal processes in the adult by promoting neuronal stem cell survival, neuron migration, and wound healing and tissue repair [1], [2].

One of the most extensive studied members of the FGF family is Fibroblast growth factor-2 (FGF2). FGF2 plays several distinctive roles in a variety of biological systems. FGF2 is a potent angiogenic molecule that *in vivo* and *in vitro* stimulates smooth muscle cell growth, wound healing, and tissue repair [3], [4]. In addition, it has been shown that FGF2 may stimulate haematopoiesis [5] and potentially plays an important role in the differentiation and/or function of the nervous system [6], the eye [7], and the skeleton [8].

FGF2 is able to interact with four different FGF receptors (FGFR1-4) [9]. The mode of receptor interaction has been matter of debate. Heparin and heparan sulfate proteoglycans (HSPGs) are able to promote FGF2 dimer/multimer formation and to modulate receptor binding. In one model, it has been proposed that heparin promotes FGF2 dimerization through direct contact between two FGF2 molecules [9], [10]. Each dimerized molecule is then able to interact with one FGFR receptor to promote its activation. In another model based on the 3D structure of the FGF/FGFR/heparin complex, it was shown that heparin/heparan sulfate constitutes a dimerization template for FGF2 monomers. These then interact with the receptor in a 2∶2∶1 (FGF/FGFR/heparin) configuration [11]. Yet in another widely accepted model, FGF2 monomers bind directly to FGFRs, which are then stabilized by heparin in a 2∶2∶2 configuration [12]. In the last twomodels, no direct contact is occurring between two FGF2 monomers.

Among the other FGF family members, FGF9 and FGF20 are known to homo-dimerize by direct interaction of each monomer and the structures of their dimers has also been solved [13]. FGF2 has also been claimed to homo-dimerize by direct monomer interaction in a few studies as indicated above [9], [10].

In this article, we investigated FGF2 dimerization using biophysical and biochemical methods and demonstrated that only heparin-affinity purified FGF2 and not FGF2 purified via ion exchange showed perturbations in the ^15^N Heteronuclear Single Quantum Coherence (HSQC) spectra indicating its ability to dimerize and to exhibit direct contact with another FGF2 monomer. This can be partially mimicked by adding heparin tetrasaccharide to ion exchange-chromatography purified FGF2. It has been assumed that only higher-order saccharides (> =  octosaccharide) promote FGF2 dimerization [14]. However, our results indicate that small heparin fragments (<octassachride) promote weak (transient) FGF2 dimers that allow direct contacts between two FGF2 monomers in opposite to higher order oligosaccharides, which, on the contrary, promote stable dimers. We also describe an assay to investigate several aspects of FGF dimerization using AlphaScreen technology, which could also be useful for the study of other FGF family members such as FGF9 or FGF20.

## Materials and Methods

### Materials

Heparin from porcine intestinal mucosa, anionic citrate and mesoglycan sulfate were purchased from Sigma. Recombinant FGF2 and PF4 were from R&D, France. Rabbit anti-FGF2 antibodies were from Santa-Cruz Ltd.

### Recombinant FGF2 protein production for biochemical studies

The cDNA encoding the 18 kDa human FGF2 form was amplified by PCR using FGF2-specific primers containing the attB adapter sequence and was cloned in the pDONR201 vector (Invitrogen) using the GATEWAY BP-reaction system (Invitrogen). Clones with the insertion were sequence-verified and used to create the final GATEWAY-expression constructs by LR cloning (Invitrogen) in GATEWAY modified pGEX-2TK (N-GST) or in pDEST42 (C-His, Invitrogen). Expression constructs were transfected into BL21(DE3)pLysS cells. Protein expression was induced at 37°C for 2–3 h by addition of 1 mM isopropyl-β- Dithiogalactopyranoside (IPTG) to exponentially growing cells. Tagged FGF2 recombinant proteins were purified by affinity chromatography using GST-HiTrap or HiTrap Heparin HP column (GE Healthcare, USA) according to the manufacturer's instructions. Eluted FGF2 was desalted and concentrated by using a Amicon Ultra-4 10 kDa centrifugal filters (Millipore, France) in 50 mM HEPES, pH 7.4.

The molecular mass and purity of the proteins were analyzed using SDS-PAGE and Western blotting with anti-FGF2 antibodies.

### FGF2 purification for NMR study

For NMR study, the ^15^N-labeled human recombinant protein FGF2 lacking of the N-terminal 9 residues was expressed in *Escherichia coli* supplemented with ^15^N-NH_4_Cl as sole nitrogen source (FGF2 cDNA coding for 18 kDa FGF2 cloned into a pET43.1a vector without tag). The bacteria grown in the M9 minimal media with ampicillin (0.1 mg/mL) at 37°C were induced by adding IPTG when OD_600_ reached 0.6. We resuspended the bacteria cells in the loading buffer of 50 mM Tris buffer, pH 7.4 and 10 mM DTT and purified FGF2 from the supernatant of cell lysate by a HiTrap Heparin HP column or HiTrap SP Sepharose column with a NaCl gradient linearly increasing from 0 M to 2 M. Eluted FGF2 was concentrated and further purified to homogeneity by using a Superdex 75 size-exclusion column.

### NMR dilution experiment of FGF2

FGF2 was reconstituted in 90% ^2^H_2_O/10% ^1^H_2_O, 10 mM DTT and 50 mM phosphate buffer at pH 5.5 with calibration compound, 2,2-dimethylsilapentane-5-sulfonic acid (DSS). The NMR ^1^H-^15^N Heteronuclear Single Quantum Coherence (HSQC) spectra were acquired on Bruker 600 MHz or Varian 700 MHz spectrometer and 25°C, recorded with 2048 and 128 complex points in w_1_(^1^H) and w_2_(^15^N) dimension with 8 scans. Concentrations of FGF2 used in the HSQC spectra were 1.5, 1.1, 0.55, 0.375, and 0.137 mM, respectively. Chemical shift perturbations were induced depending on the conversion between dimer and monomer formation under different concentrations. We measured the chemical shift perturbations between the samples with different concentrations. The combined chemical shift perturbations were represented by [(Δδ_NH_^2^ + Δδ_N_^2^/25)/2]^1/2^, where Δδ_NH_ and Δδ_N_ are the chemical shift changes of backbone amide proton (NH) and amide (N), respectively [15]. The backbone resonances of free FGF2 were characterized based on known chemical shift assignments (BMRB accession number 4091) [16], [17]. Due to the fast exchange binding regimes, the backbone of the chemical shift movements were traced in the dilution experiments.

### AlphaScreen technology

AlphaScreen of FGF2 dimerization was carried out with FGF2-N-GST, FGF2-C-His according to the manufacturer's indications (PerkinElmer, Inc.) and as described earlier [18]. Briefly, Reaction mixtures were prepared in 20 µl final volume in 384-well plates. Firstly, 5µl of each prepared dilutions of FGF2-N-GST and FGF2-C-His in the AlphaScreen^™^ reaction buffer (50 mM Hepes pH 7.4, 150 mM NaCl, 5 mM MgCl2, 1 mM DTT, 0.1% BSA and 0.05% Tween-20) were incubated together for 30 min at room temperature. Subsequently, 5 µl of AlphaScreen^™^ Glutathione Donor beads and 5 µl of Ni Chelate Acceptor beads (PerkinElmer, Inc., 25 µg/ml final concentration) were added to the mix and the plate was further incubated for 1 h at room temperature before signal measurement. When a competition assay was performed, 5 µl of a competitor (at various concentrations) were added to the mix for 30 min at room temperature before incubation with the beads. Plates were read on EnVision 2103 Multilabel Plate Reader (PerkinElmer, Inc.) equipped with AlphaScreen^™^ optical detection module.

IC_50_ values were determined using a sigmoidal dose-response (variable slope) equation (Graphpad Prism, San Diego, CA).

### Cross-linking of FGF2

The amine-specific homobifunctional cross-linker bis(sulfosuccinimidyl) substrate (BS3; Pierce Biotechnology, USA) was used to cross-link FGF2 according to the protocol modified from Perollet et al. [19]. The recombinant FGF2-C-His was labeled with IRDye 800CW Protein Labeling Kit–Low MW (LICOR Biosciences, Lincoln, NE) according to the manufacturer's instruction. The labeled FGF2-IRDye conjugate was characterized with a dye to protein ratio of 3∶3. The labeled FGF2 in 50 mM HEPES was incubated for 1 h at room temperature and after incubated for 30 min with 1 mM freshly prepared BS3 solution. Each reaction mixture was quenched with 1 M Tris HCl at pH 8.0. Cross-linked samples were analyzed by 10% SDS-PAGE. The IR signal was visualized using Odyssey Infrared Imaging System (LICOR Biosciences).

### Solid-phase ligand-binding assay

The assay was performed as described earlier [19]. Briefly, 500 nM FGF2-C-His was immobilized in 96-wells high-binding plates and 50 nM FGF2-N-GST in the presence or absence of 1 µg/ml heparin was added. The complex was revealed with anti-GST antibody and secondary HRP conjugated antibodies. After adding peroxidase substrate TMB (Sigma-Aldrich, the absorbance was measured at 450 nm in a microplate reader.

### Cell proliferation

Endothelial cells (Human Dermal Lymphatic Endothelial Cells (LEC), Promocell, France), were grown in Endothelial Growth Medium MV (EGM MV, Promocell) supplemented with 5% fetal bovine serum (FBS) and growth factors according to the manufacturer's instructions at 37°C in a 5% CO_2_ atmosphere. LEC (3000cells/well) were plated in 96-well plates in EGM MV. After 24 h, the cells were starved in the medium supplemented with 0.3% FBS for 7 h and then the indicated concentration of FGF2 or mutants were added for 72 h. Cell proliferation was quantified by Cell Proliferation Reagent WST-1 (Roche).

### Replicates

All results reported are performed in, at least, two independent assays done in triplicates or quadruplicates. Data are expressed as mean ± SEM.

## Results

### Biophysical measurement of FGF2 dimerization from FGF2-purified with or without heparin-affinity

**Figure 1:**
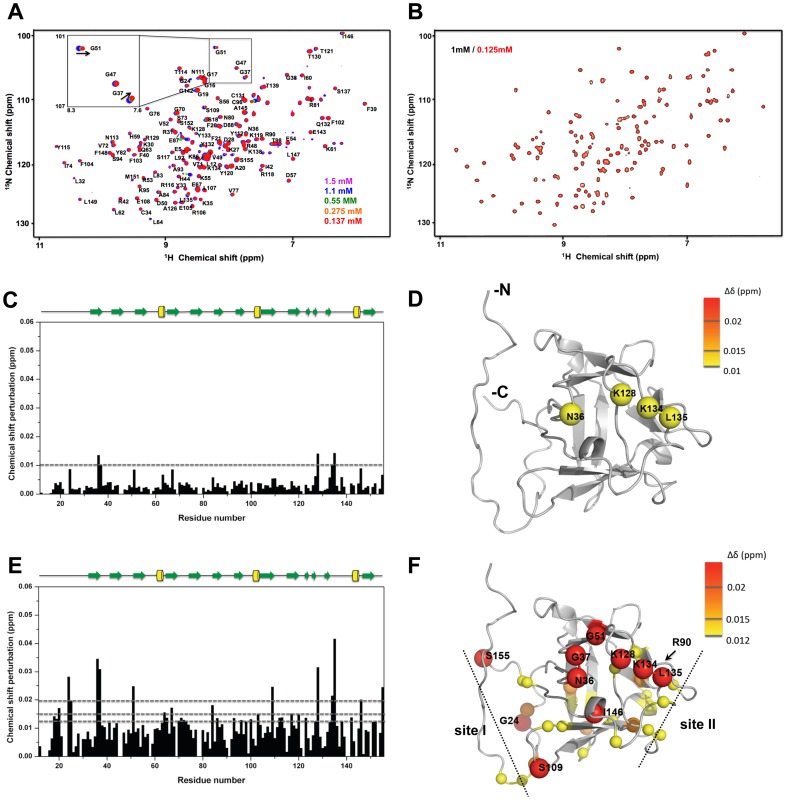
Chemical shift perturbation upon variation in FGF2 concentrations. A. HSQC spectra of heparin column purified FGF2 of 1.5 mM (purple), 1.1 mM (Blue), 0.55 mM (green), 0.275 mM (orange) and 0.137 mM (red). B. HSQC spectra of ion-exchange column purified FGF2 of 1 mM (black) and 0.125 mM (red). Chemical shift difference is only observed in the heparin-column purified FGF2 and the difference is analyzed by the combined chemical shift perturbation, [(Δδ_NH_^2^ + Δδ_N_^2^/25)/2]^1/2^. C. Chemical shift perturbation of the heparin-column purified FGF2 is between 0.137 mM and 0.55 mM and (D) the residues with perturbation larger than the threshold value of 0.01 ppm were highlighted on the FGF2 structure (PDB code: 1BLA) with yellow spheres. E. Chemical shift perturbation of the heparin-column purified FGF2 between 0.137 mM and 1.5 mM and (F) the residues with perturbations larger than the threshold values of 0.012, 0.015 and 0.02 ppm were pointed out FGF2 structure as yellow, orange and red spheres, respectively where the radius of sphere reflects the quantity of chemical shift perturbation. The proposed two dimer interfaces are indicated by dash lines.

FGF2 purified with or without heparin-sepharose chromatography exhibits different profiles in NMR HSQC dilution experiments (Fig. 1). Differences in the chemical shift between samples at various concentrations were observed for heparin-affinity purified FGF2 (Fig. 1A). No differences are detected for ion exchange purified FGF2 (Fig. 1B). This may be due to the different purification methods used for both FGF2s. FGF2 purified with heparin-sepharose chromatography is likely to contain trace contamination of heparin stripped from the resin during the elution step, which is not the case for FGF2 purified by ion-exchange chromatography. These trace amounts of heparin appear to modulate the oligomerization state of FGF2 in solution for different FGF2 concentrations. Therefore, we monitored the differences in chemical shift for FGF2 concentrations ranging from 0.137 mM to 1.5 mM. The chemical shift perturbation reveals two sequential effects in the dilution experiments. First, when samples of 0.137 mM and 0.55 mM are compared, four residues (N36, K128, K134 and L135) showed the most significant perturbations in the HSQC spectra (Fig. 1C and D). These perturbed residues correspond to the heparin-binding residues as reported elsewhere [20]. This suggests that increasing FGF2 concentrations enhance FGF2-heparin complexes. Second, when sample with the lowest and the highest concentration are compared, the number of perturbed residues is proportional to the concentration. These were not limited to the heparin-binding site (Fig. 1E and F). One additional perturbed region is located near the C terminal surface consisting of G24, S109 and S155 that exhibited significant chemical shift perturbations (> 0.02 ppm) (Fig. 1E). Previous literature reported that the two residues (S109 and R90) located at the opposite locations in FGF2 are involved in dimer contact interface [10], [21]. Therefore, the C-terminal surface containing S109 might act as the primary dimer interface (site I in Fig. 1F). Another perturbed site includes residues with lesser perturbations (> 0.012 ppm) including A84, K86, A93, S96 and E100 that are located at ß6, the loop between ß6 and ß7, ß7 and α2 respectively. These residues are located in the proximity of R90. Therefore, they constitute a secondary dimer interface (site II in Fig. 1F). On the contrary, no chemical shift perturbations were detected for FGF2 only purified by ion exchange chromatography.

**Figure 2:**
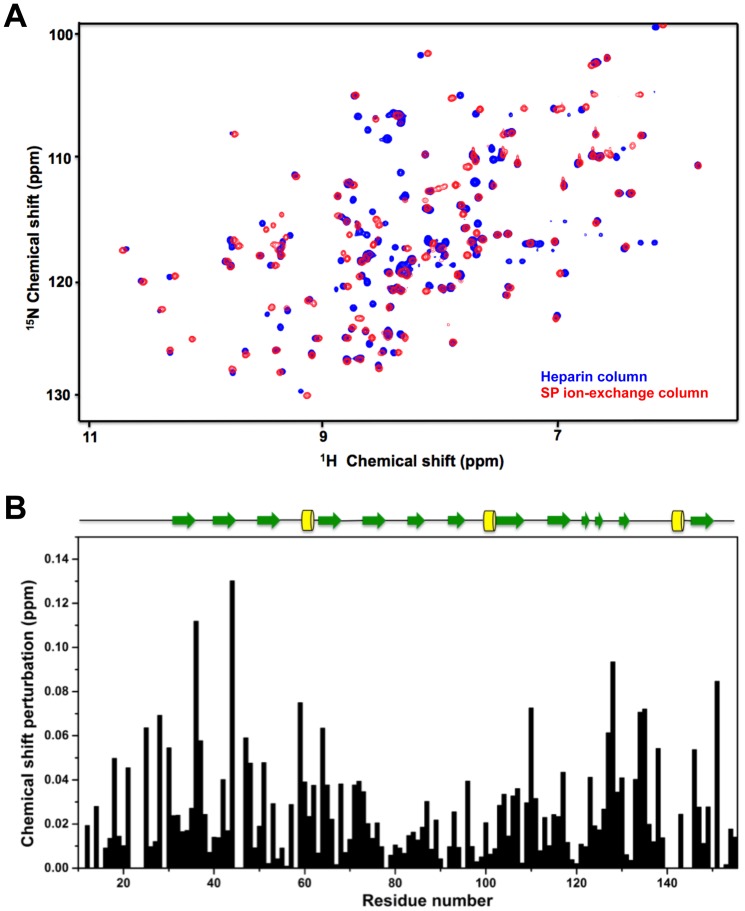
Comparison of HSQC spectra purified by two different methods. (A) Superposition of HSQC spectra of heparin-affinity purified (blue) and ion-exchange purified FGF2 (red). (B) Chemical shift differences between the two spectra analyzed by the combined chemical shift perturbation are indicated.

We compared the HSQC spectra of FGF2 that were purified by the two different methods (Fig. 2A). We noticed significant differences between the two spectra. There are ∼50% residues with different chemical shift patterns in the two spectra (Fig. 2B). Significant perturbations are mainly located at the residues near the N- and C-terminal portions. The residues at the C-terminus constitute the proposed heparin-binding site. Thus, these differences are in favor of the presence of stripped heparin fragments that modify the HSQC spectra of heparin-affinity purified FGF2. In addition, the observed differences indicate the presence of a FGF2 dimerization interface (similarity between Figure 2B and 1E).

Taken together, the perturbation observed for heparin-affinity purified FGF2 at different concentrations revealed a heparin binding site and two dimer interfaces. Higher protein concentration forces FGF2 monomers to establish contacts with each other to form dimers or higher order oligomers. FGF2 purified by ion-exchange chromatography did not show similar perturbation. This indicates that self-association of FGF2 is eliminated if purification is done without heparin affinity chromatography.

**Figure 3:**
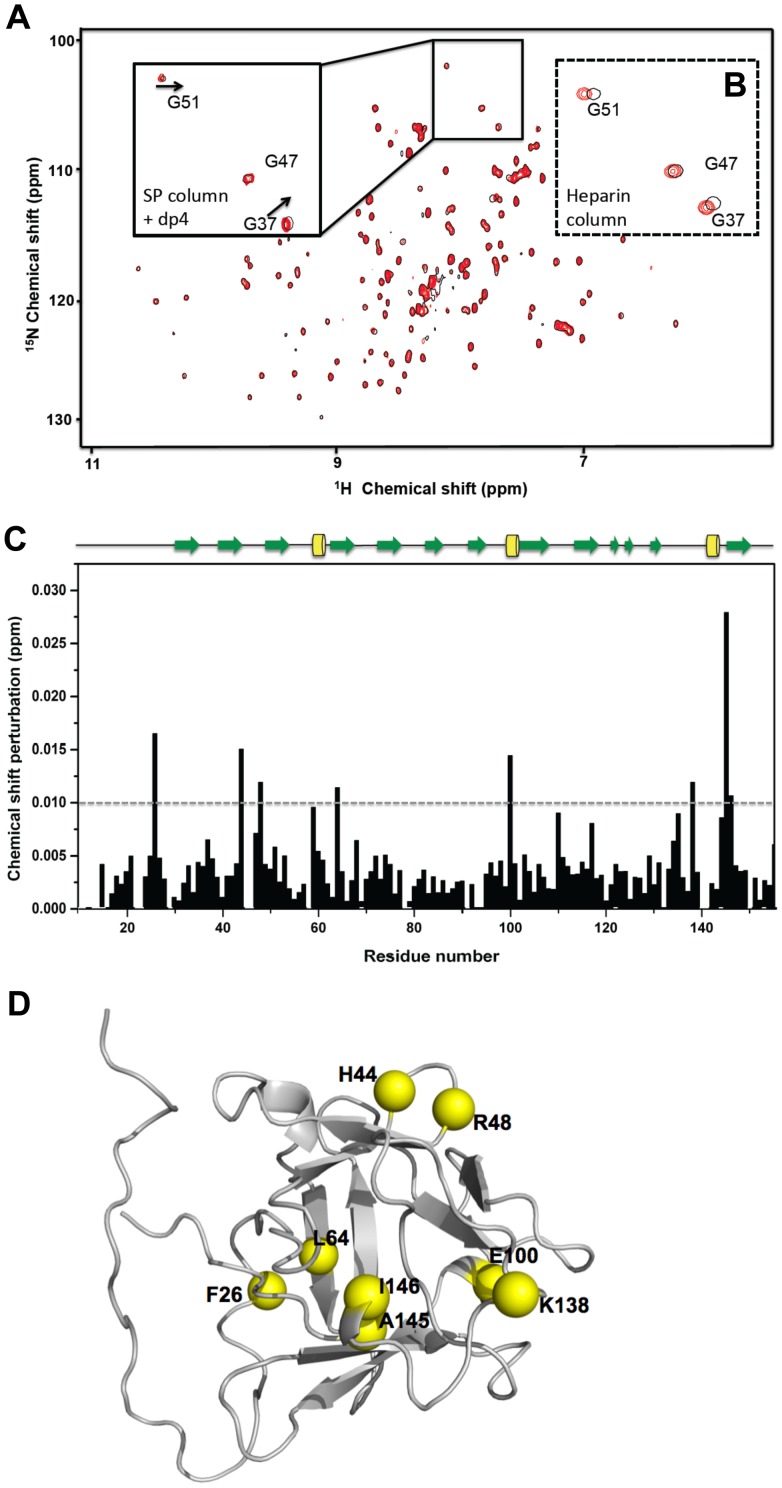
Chemical shift perturbation upon changing FGF2 concentrations in the presence of heparin tetrasaccharide (hep-4). (A) HSQC spectra of FGF2 in complex with hep-4 with molar ratio of 1: 0.5. FGF2 at 0.125 mM (black) and 1.0 mM (red). The inset shows the close view of resonances of G47 and G51. (B) The corresponding close view of HSQC spectra of heparin column purified FGF2: 0.137 mM (black) and 1.5 mM (red). (C) Chemical shift perturbation of FGF2/hep-4 complex for FGF2 at 0.125 mM and 1.0 mM. (D) The residues in FGF2 with perturbations larger than the threshold values of 0.01 ppm are indicated as yellow spheres.

We next investigated whether heparin fragments when added to affinity purified FGF2 were able to induce similar changes. We purified heparin fragments and added the tetrasaccharide (hep-4) to ion-exchange chromatography purified FGF2. We performed the similar dilution experiment under the condition of molar ratio 1: 0.5 for FGF2 to hep-4 and chemical shift difference were monitored with FGF2 concentrations ranging from 0.125 mM to 1 mM (Fig. 3). Similar to heparin-column purified FGF2, we also detected comparable resonance shifting with increasing FGF2 concentration. Representative close views of the corresponding HSQC spectra are compared in Figure 3A where two selected resonances of G47 and G51 demonstrated similar movements between the two cases. We saw similar dilution effects. Comparing to the dilution-induced chemical shift modifications in Figure 1E, some differences were observed (Fig. 3B). We observed less perturbation and a slightly shifting of the perturbed region. This indicates that heparin fragments stripped from the column during elution are not fully identical to the tetrasaccharide. However, the concentration-dependent effect can be reproduced to some extend by adding short-chain heparin. Interestingly, we did not see the effect when we diluted the samples containing FGF2-heparin dodecasaccharide (hep-12) complexes. We suspect that the binding between FGF2 and hep-12 is too strong to be dissociated when the concentration is decreased to 0.1 mM. Thus, no significant chemical shift perturbations could be detected in the FGF2-hep12 dilution experiment (Fig. S1).

### Further characterization of FGF2 dimerization of heparin-affinity purified FGF2

#### FGF2 dimerization in C-N orientation

**Figure 4:**
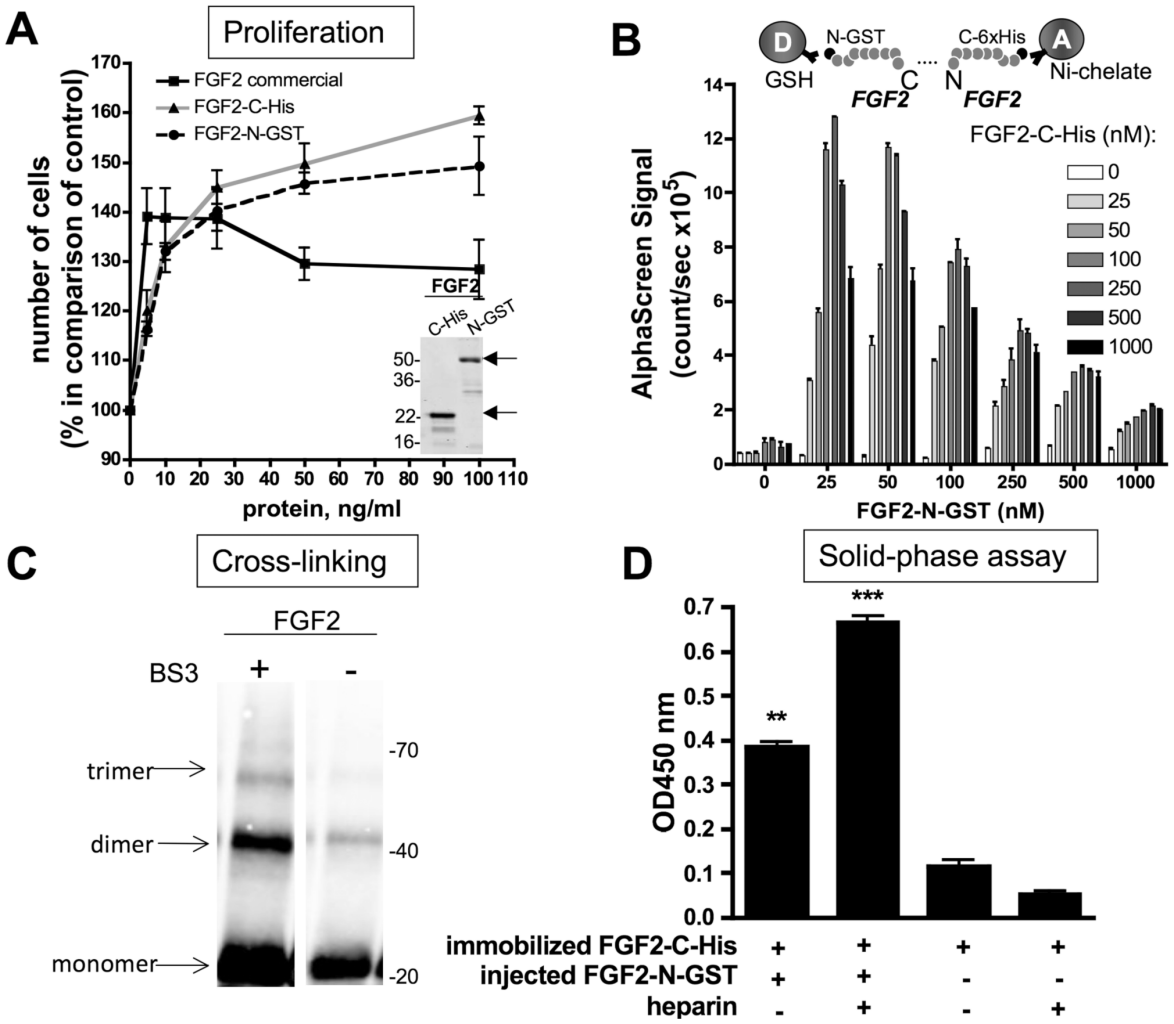
FGF2 dimerization in C-N orientation. A. Dose-dependent proliferation of blood endothelial cells induced by recombinant FGF2-C-His and FGF2-N-GST as well as commercial FGF2. Proliferation of cells grown for 72 h was determined by Cell Proliferation Reagent WST-1. One representative experiment is shown, values are as the mean ± SEM (n = 4). *In the inset*, recombinant FGF2-C-His and FGF2-N-GST were analyzed by 10% denaturating SDS-PAGE. Protein levels were visualized by Coomassie staining. Molecular weight marker is indicated on the left. B. Detection of FGF2 dimerization by AlphaScreen^™^. Top panel: assay design of the AlphaScreen^™^ experiment. FGF2-N-GST was bound to AlphaScreen^™^ Glutathione Donor beads and FGF2-C-His to AlphaScreen^™^ Ni chelate acceptor beads. Bottom panel: Direct interaction between FGF2-N-GST and FGF2-C-His. The recombinant proteins were incubated at indicated concentrations with donor and acceptor beads for 24 h at room temperature before signal measurements. Histograms are representative of three independent experiments with comparable results. Data are expressed as mean ± SEM (n = 4). C. Cross-linking assay of FGF2. 1µg FGF2 labeled with near-infrared fluorescent IRDye800CW (LICOR Biosciences) was incubated with or without the cross-linker BS3. Cross-linking samples were analyzed by 10% SDS-PAGE under reducing conditions. The IR signal was visualized using Odyssey Infrared Imaging System (LICOR Biosciences). The arrows show cross-linked FGF2 oligomers. Molecular weight marker is indicated on the right. D. A solid-phase ligand-binding assay with immobilized 500 nM FGF2-C-His and 50 nM soluble FGF2-N-GST in the presence or absence of 1µg/ml heparin. The dimerization was revealed as described in the Material and Methods. Representative experiment was done in duplicate. Error bars represent the mean ±SEM (n = 4).

FGF2 dimerization in the absence of heparin or modulators:

We used an amplified luminescence proximity homogeneous assay (AlphaScreen) to detect and measure dimer/oligomer formation of heparin-affinity purified FGF2 [18]. To configure the AlphaScreen for monitoring FGF2 interactions, recombinant tagged proteins for FGF2 were used. Glutatione S-transferase (GST) or His-tagged FGF2s were expressed in *E. coli*, purified on GST or Nickel columns respectively, followed by heparin affinity chromatography. The purity of FGF2 as well as its biological activity was assessed by its capacity to stimulate endothelial cell proliferation in comparison with a commercial FGF2, which was similar in both cases (Fig. 4A).

N-terminal GST tagged FGF2 and C-terminal 6xHis tagged FGF2 were bound to glutathione donor beads and Ni-chelate acceptor beads respectively. Their respective signals were detected by the Alphascreen assay (Alphascreen signal in count per minute: cpm). This allows FGF2 to dimerize in a configuration where the C-terminus and the N-terminus of FGF2 can interact. The maximal signal for the FGF2-N-GST:FGF2-C-His interaction was reached at 25-50 nM of FGF2-N-GST and 100-250 nM of FGF2-C-His followed by a decrease of the AlphaScreen signal to background levels (Fig. 4B). This trend was reproduced with two different protein batches. The hooking effect (biphasic behavior) is likely due to saturation of all available binding sites on the beads [22].

The specificity of the interaction has already been investigated. For the control, we performed an AlphaScreen ^™^ experiment by using FGF2 and the unfolded response protein IRE1. In this case, His-tagged IRE1 is bound to the Ni chelate acceptor beads and FGF2-GST bound to gluthatione donor beads. No detectable signal was evident [23].

FGF2 dimerization was confirmed by two alternative methods. First, cross-linking in solution of FGF2-C-His revealed a strong signal for dimerized FGF2 and a weaker signal for a multimeric complex when incubated with the cross-linking agent BS3 (Fig. 4C). Second, a solid-phase ligand-binding assay demonstrated binding of soluble FGF2-N-GST to immobilized FGF2-C-His. The dimerization signal is increased in the presence of heparin (Fig. 4D).

**Figure 5:**
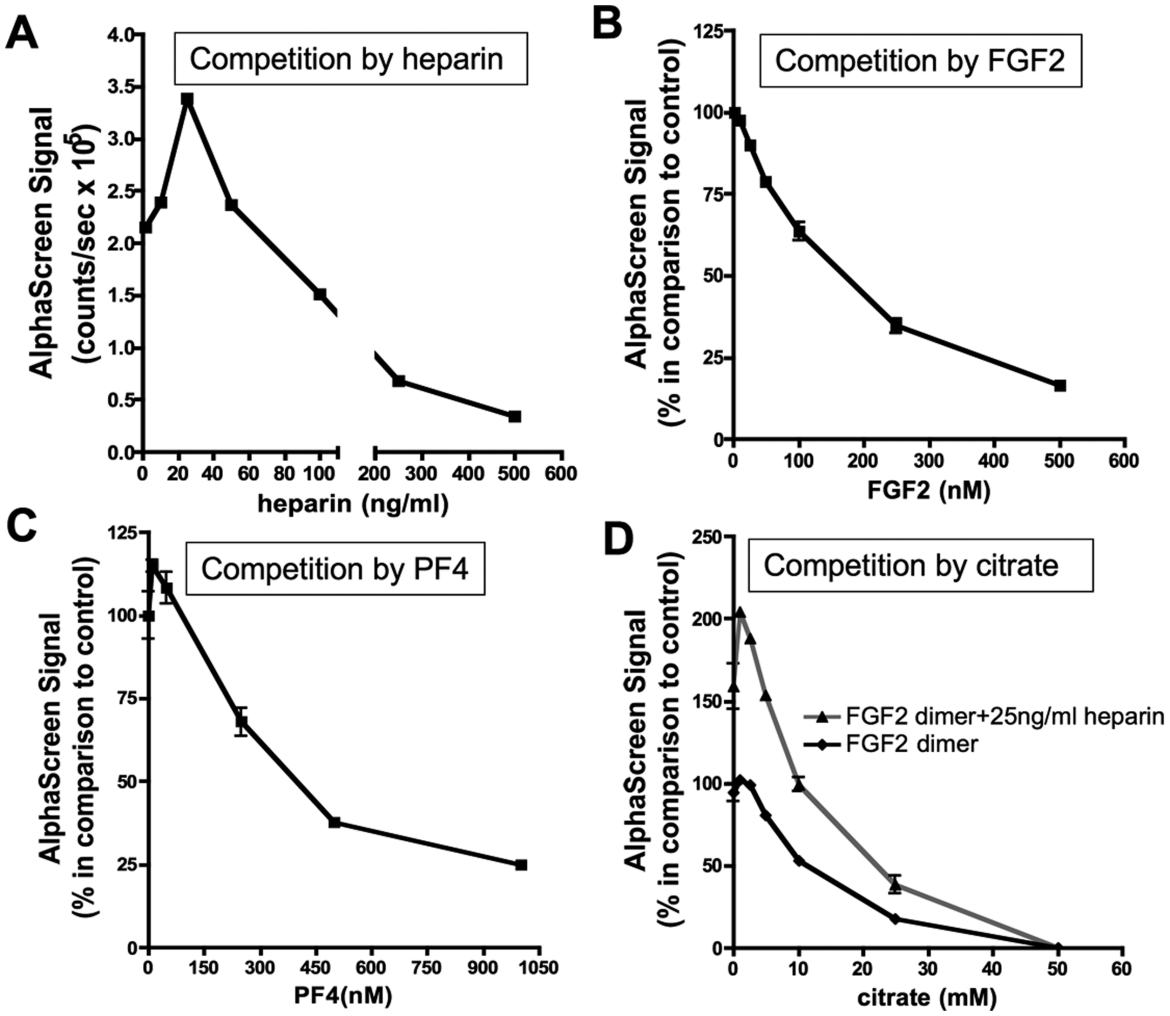
Competition assay to assess FGF2 dimerization (C-N orientation) using the AlphaScreen^™^ technology. A. Competition assay of FGF2 dimerization with heparin-like substances. The interaction between 50 nM FGF2-N-GST and 50 nM FGF2-C-His *was* competed in the presence of increasing concentrations of heparin. Tag less FGF2 (B) and PF4 (C) compete for FGF2 dimerization of 50 nM FGF2-N-GST and 50 nM FGF2-C-His. D. Anionic citrate inhibits heparin-dependent and independent FGF2 dimerization. (A through D) AlphaScreen^™^ signal is expressed as percentages in comparison of control; AlphaScreen^™^ signal corresponding to FGF2 dimer without competitors is set as 100%. Results are representative of at least three independent experiments. Results are mean values ± SEM (n = 3).

Modulators of FGF2 dimerization and their effects:

We then carried out competition experiments to further validate our AlphaScreen^™^ assay. In our assay, FGF2-N-GST:FGF2-C-His was used at the optimal concentration of 50 nM:50 nM to be able to detect either an increase or a decrease in signal. Heparin showed a biphasic dose-dependent effect on FGF2 dimerization (Fig. 5A). The maximum signal for the FGF2 dimer was detected at low concentration of standard heparin (5 ng/ml) with a decrease at higher heparin concentrations (IC_50_ value of 98.7 ng/ml) (Fig. 5A). The specificity of the FGF2 dimerization was confirmed in a competition assay. In the presence of tag-less FGF2, the AlphaScreen signal demonstrated a concentration-dependent decrease with an IC_50_ value of 180.3 nM (Fig. 5B). Inhibition of the FGF2 dimerization signal was also observed with platelet factor 4 (PF4) with an IC_50_ value of 265.1 nM (Fig. 5C). This result is in agreement with published data showing that PF4 is an inhibitor of FGF2 dimerization [19].

We next used anionic citrate as an inhibitor, and investigated its effect in the AlphaScreen assay. Citrate mediates the monomer-dimer equilibrium of many proteases for the regulation of their catalytic activity [24], [25]. We wondered whether citrate could also have an effect on FGF2 dimerization. We observed an inhibition effect of citrate in heparin-induced and non-induced FGF2 dimerization with IC_50_ 11 mM (fig. 5D).

#### FGF2 dimerization in the N-N orientation

FGF2 dimerization in absence of heparin or modulators:

We next analyzed FGF2 dimerization in the N-N configuration using AlphaScreen. On the contrary to the experiments described above, 6xHis tagged on C-terminus FGF2 was exclusively used and bound to both Ni-chelate acceptor and donor beads leaving N-termini free for interaction.

**Figure 6:**
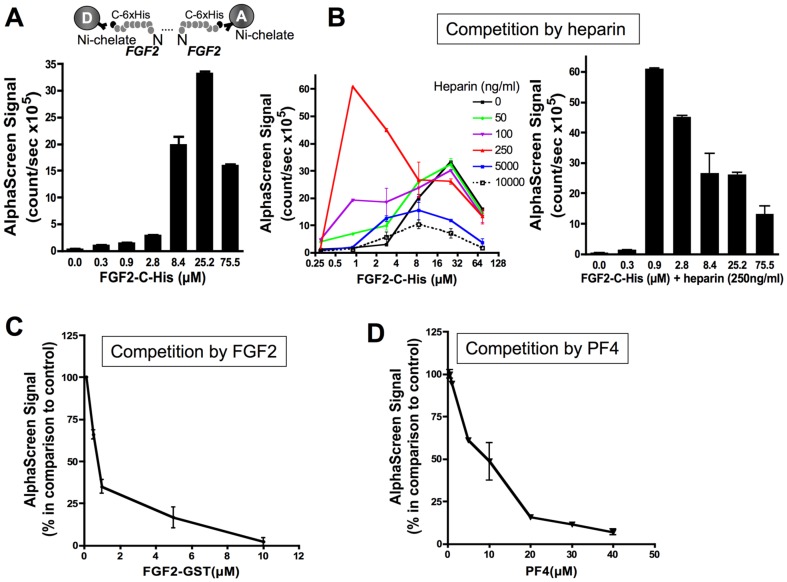
FGF2 dimerization and competition in the N-N orientation. A. Top panel: assay design of the AlphaScreen^™^ experiment. FGF2-C-His was bound to AlphaScreen^™^ Ni chelate donor and acceptor beads. Bottom panel: For dimerization, increasing amounts of FGF2-C-His were incubated with the beads for 24 h before AlphaScreen^™^ signal measurements. B. Competition assay of FGF2-C-His dimerization at various concentrations of FGF2 with increasing concentrations of heparin (left). FGF2 dimer formation in the presence of 250 ng/ml heparin (right). FGF2-GST (C) and PF4 (D) compete for 1µM FGF2-C-His dimerization. (A through D) Results are representative of at least three independent experiments. Results are mean values ± SEM (n = 3).

AlphaScreen was first carried-out using increasing amounts of FGF2-C-His (0–75.5 µM) (Fig. 6A). A maximal AlphaScreen signal reflecting the direct interaction between two FGF2-C-His molecules was reached at concentrations of 25.2 µM FGF2 with an EC_50_ value of 6.35 µM. We next investigated whether exogenous FGF2 was able to compete with FGF2 dimerization in this assay. When 1 µM His-tagged FGF2 was used for immobilization on acceptor and donor beads, exogenous FGF2 competed efficiently in the AlphaScreen with an IC_50_ value of 0.62 µM (Fig. 5C).

FGF2 dimerization in the N-N configuration in the presence of exogenous heparin or modulators:

As for the C-N configuration experiments, exogenously added heparin was able to modulate FGF2 dimerization in the N-N configuration. In this configuration, different concentrations of heparin increased transitorily the AlphaScreen signal at various FGF2 concentrations and decreased it afterwards (Fig. 6B). At a heparin concentration of 250 ng/ml, the peak for the AlphaScreen signal was right-shifted with respect to heparin concentrations (Fig. 6B). This indicates that heparin facilitates FGF2 dimer formation at lower FGF2 concentrations. As in the C-N configuration, PF4 inhibited FGF2 dimerization in the N-N configuration with a IC_50_ value of 9.56 µM (Fig. 6D).

These data indicate that FGF2 can dimerize in two different configurations in the AlphaScreen^™^ assay. The C-N configuration of FGF2 seems to be more favourable since the maximal Alphascreen signal was reached with lower FGF2 concentrations comparing to FGF2 dimerization in the N-N configuration. Exogenously added heparin modulated the level of dimerization in all orientations. PF4, a known FGF2 inhibitor, and citrate also reduce FGF2 dimerization.

## Discussion

FGF2 is one of the most potent mitogenic and proangiogenic factors. FGF2 mediates its biological activity by binding to specific cell-surface high-affinity tyrosine kinase receptors and heparin-like glycosaminoglycan [3], [4]. It has been reported that FGF2 is able to dimerize [9]–[11], [21], [26]. FGF2 dimerization has been described to occur in solution without heparin but can be significantly increased in the presence of heparin or HSPGs [9]–[11], [21]. Oligomerization has also been observed and it depends critically on heparin or heparan sulfates.

In this article, we show that only heparin-affinity purified FGF2 but not FGF2 purified without heparin is able to dimerize. Furthermore, we described an assay to measure FGF dimerization using AlphaScreen^™^, which allows the detection of interactions between (two) biological partners immobilized on acceptor and donor beads.

We initially determined the different profiles in NMR HSQC dilution experiments of heparin-affinity purified FGF2 and FGF2 purified without heparin affinity chromatography. HSQC spectra for heparin-affinity purified FGF2 revealed four residues (N36, K128, K134 and L135) with the most significant perturbations, and corresponding to the heparin-binding site [20]. This is in favour of the presence of trace amounts of heparin in the sample after a heparin-column purification. With increasing concentrations, direct contact between heparin-affinity purified FGF-2 monomers was promoted with the appearance of two additional perturbed sites.

One can only speculate why self-association is promoted when FGF2 is purified via heparin sepharose chromatography. Small heparin fragment may be removed from the heparin sepharose column during elution of FGF2. This may trigger an initial step where two FGF2 monomers are brought in close proximity. Each FGF2 monomer is then, in a second step, able to associate which each other. FGF2 purified with ion-exchange chromatography, which is devoid of potential heparin contamination, did not show similar perturbations.

To test whether exogenously added heparin fragments were able to elicit similar changes, we performed the HSQC experiment using a heparin tetrasaccharide, which was added to ion-exchange purified FGF2. It has been shown that small heparin fragments can induce FGF2 multimers [21], [14]. In this case, comparable resonance shifting with increasing FGF2 concentration was also detected. Nevertheless, some differences in the chemical shift profile were still observed, which indicate that additional molecular interactions are involved. To our surprise, we did not see the effect when heparin dodecasaccharide (hep-12) was used. These observations also suggest that small heparin fragments can induce perturbations in HSQC spectra that reflect direct contact between two FGF monomers. Thus, trace amount of heparin enables self-association of FGF2 to some extent. These data also indicate the small heparin fragments may promote direct contact between two FGF2 monomers. However, it is not clear how stable the self-association is and to what extent this may impact on cell signalling. The strength of association between heparin and FGF2 is dependent on chain-length and degree of sulfation of heparin. In fact, Gallagher and collaborators have found differences in the effect of various heparin fragments [14]. In their study, they showed that tetra or hexasaccharides only promote 1∶1 complex (FGF2:heparin) formation whereas higher-order saccharides (> =  octosaccharide) promoted 2∶1 (FGF2:heparin) complexes when analyzed by gel filtration. This would indicate that only octasaccharides or higher-order saccharides are able to promote dimerization. However, tetrasaccharides and hexasaccharides are still able to promote mitogenesis. In their model, no direct contact between two FGF2 monomers is postulated. This seems in contradiction with the results we report herein. However, HSQC is a much more sensitive method than gel filtration and is able to detect weak and transient interactions between molecules. Furthermore, the HSQC results we provide clearly indicate perturbations that are only explained by a direct interaction between two FGF monomers. In our opinion and based on the HSQC analysis, small heparin fragments may promote weak (transient) FGF2 dimers that allow direct contacts between two FGF2 monomers. They may not be enough stable to be evidenced by gel filtration. In fact, analytical ultracentrifugation (AUC), we performed, also evidenced mainly FGF2 monomers, which is in agreement with the latter data. Higher order oligosaccharides, on the contrary, may promote stable dimers that do not necessarily involve direct contact between two FGF monomers and that are detectable by gel filtration and AUC.

In this article, we have also developed a simple method to study dimerization of heparin-affinity purified FGF2 further. To this aim, recombinant FGF2 was produced as C-terminal or N-terminal tagged GST or His proteins and further purified by heparin-affinity chromatography. Tagged proteins were then bound to acceptor or donor beads and dimerization studied in either the C-N or N-N configuration by AlphaScreen^™^. These experiments were only conducted with heparin-affinity purified FGF2, since only in this case chemical shift perturbations were observed. The maximal AlphaScreen^™^ signal reflecting the direct interaction between two immobilized FGF2s obtained for the C-N configuration was reached at much lower FGF2 concentrations as for FGF2 in the N-N orientation. This indicates that the C-N orientation is more favourable for FGF2 dimerization. Furthermore, our NMR dilution experiment showed that the two distinct dimer interfaces are located at two different sides of FGF-2. We also confirmed the model that two FGF-2 molecules bind to one heparin molecule and align asymmetrically in a head-to-tail fashion in a *cis* configuration [10].

The specificity of FGF2 dimerization in both configurations was confirmed in two ways. As demonstrated previously, FGF2 did not interact with IRE1 when this protein was used as a control [23]. Furthermore, FGF2 added exogenously into the assay fully competed for dimerization in a concentration-dependent manner.

The FGF dimerization assay was further validated using known molecules able to interact with FGF2. Heparin exhibited a biphasic dose-dependent effect on FGF2 dimerization. The AlphaScreen signal peaked at low heparin concentrations followed by a decrease with increasing heparin concentrations. This behaviour is in agreement with the literature showing that the formation of FGF2 dimer is dependent on the ratio between heparin and FGF2 [27].

We further demonstrated that PF4 was able to inhibit FGF2 dimerization in the AlphaScreen assay. This is in agreement with previously published observations [19], [28]. Indeed, PF4 has been shown to associate with FGF2 in a 1∶1 complex using a variety of biochemical and physico-chemical approaches. Moreover, we also demonstrate that citrate affects on FGF2 dimerization. Citrate is known to regulate the monomer-dimer equilibrium of many proteases as well as of the membrane phosphatidylinositol 3-phosphate (PIP)1-binding protein Hrs [24], [29]. Furthermore, the dimerization of other proteins such as the CTX A3 cardiotoxin from the Taiwan cobra has also been reported to be promoted by citrate [25].

Taken together, we demonstrate that only FGF2 purified by heparin-sepharose chromatography is able to dimerize and to establish heparin-dependent and heparin-independent contacts with each other. This may be due to trace amounts of heparin-molecules that favour monomer to monomer interactions. These results also indicate that FGF2 is not forming a dimer via direct contact when purified without heparin-affinity chromatography. Thus, heparin binding may represent the first critical step that subsequently will lead to direct contact between two FGF2 monomers. Furthermore, we have also described and validated in this article a new assay for FGF dimerization, which may be useful to study of the mechanisms of FGF dimerization and to develop a specific AlphaScreen-based screening tools. This assay is also suitable to conduct researches and validate chemical compounds and libraries that interfere with FGF dimerization. This may be of particular interest to other FGF family members such as FGF9 or FGF20, which are able to dimerize without the help of heparin [13], [30].

## Supporting Information

Figure S1 **HSQC spectra of FGF2 in complex with hep-12 with a molar ratio of 1: 0.5 (FGF2:Heparin).** FGF2 1.0 mM (red) and 0.25 mM (black) and FGF2 without hep-12 (blue).
(PDF)
