# Supplementary material for: Dimerization Capacities of FGF2 Purified with or without Heparin-Affinity Chromatography
Source: PLoS One. 2014 Oct 9;9(10):e110055. doi: 10.1371/journal.pone.0110055 (PMC4192534; doi:10.1371/journal.pone.0110055)
Supplement: Figure S1 — HSQC spectra of FGF2 in complex with hep-12 with a molar ratio of 1: 0.5 (FGF2:Heparin). FGF2 1.0 mM (red) and 0.25 mM (black) and FGF2 without hep-12 (blue). (PDF) [file pone.0110055.s001.pdf]

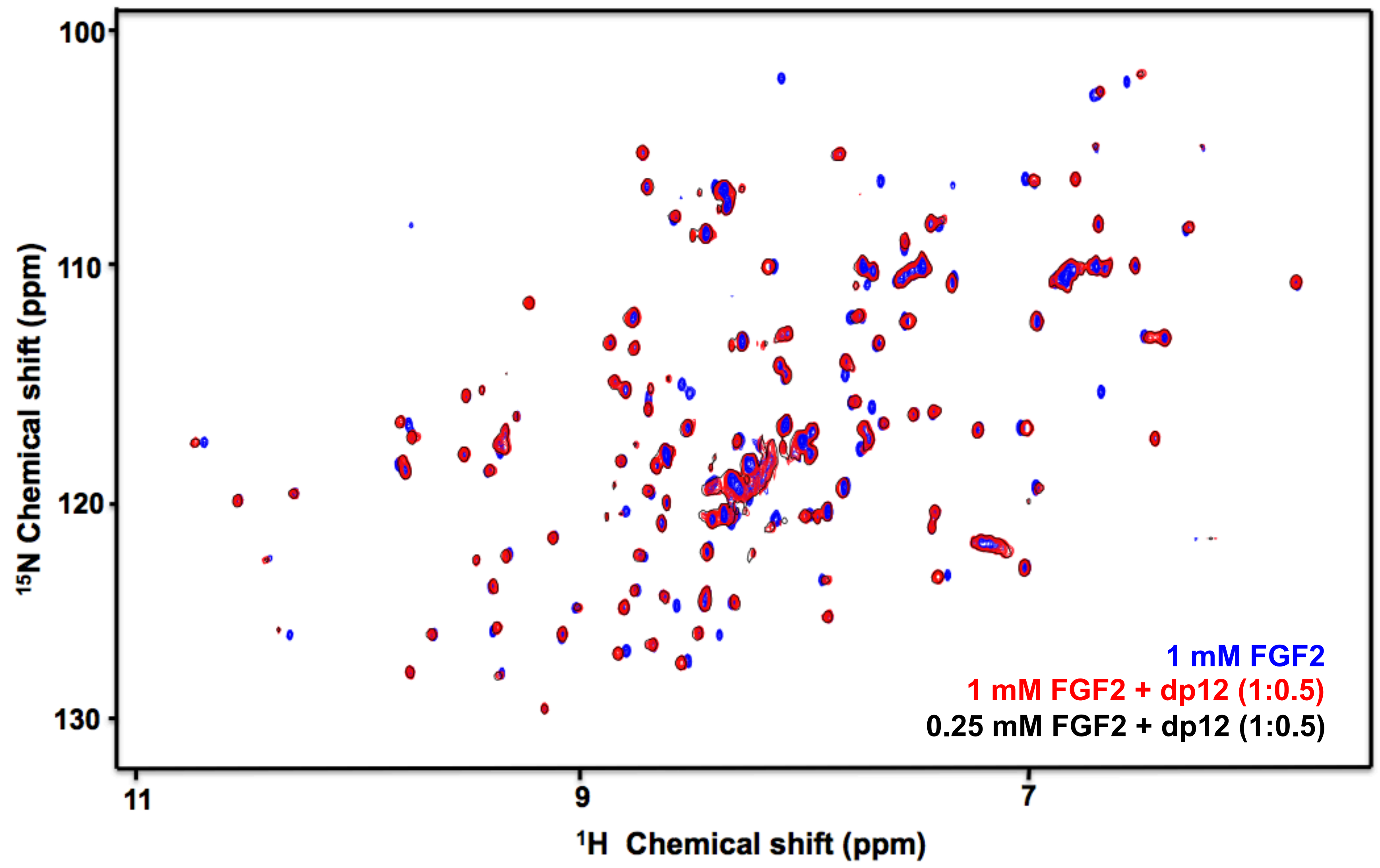

Supplemental Figures

Figure S1: HSQC spectra of FGF2 in complex with hep-12 with a molar ratio of 1: 0.5 (FGF2:Heparin). FGF2 1.0 mM (red) and 0.25 mM (black) and FGF2 without hep-12 (blue).
